# The Examination of Hydrated Cement Paste Made of CEM III/A 42,5 N-LH/HSR/NA under the Influence of Urea Solution

**DOI:** 10.3390/ma13214984

**Published:** 2020-11-05

**Authors:** Barbara Słomka-Słupik

**Affiliations:** Faculty of Civil Engineering, Silesian University of Technology, Akademicka 5 Str., 44-100 Gliwice, Poland; barbara.slomka-slupik@polsl.pl

**Keywords:** concrete corrosion, urea, decalcification

## Abstract

The production of urea, used inter alia in agriculture, is increasing. Therefore, urea hydrolysis products are expected in groundwater. Due to lack of new research on the influence of urea on the technical condition of concrete structures, the changes that this compound may cause to hardened cement paste were initially check. After 11 months of immersion of hardened cement paste in 20% CO(NH_2_)_2_ solution, tests were conducted at different depths of penetration. A pH of 11.97 was recorded in the first layer with a thickness of 0.5 mm, and the pH of the innermost layer was 12.48. The decalcification process and the formation of predominantly secondary calcite in the edge layers were confirmed using XRD, SEM, and analytical methods. No nitrogen phase was formed, but the deeper was the layer, more wollastonite was present. Moreover, up to a depth of about 20 mm, the sample was mechanically weak-breakable by the force of the hands. The examination of the filtrate’s conductivity, leachable calcium content, and pH along the way of urea diffusion confirmed changes in the examined material. When analyzing the technical condition of concrete treated with urea, pH could be an indicator due to the possibility of buffer reactions.

## 1. Introduction

Urea whose systematic IUPAC name is carbonyl diamide, is an organic compound [1]. Urea is a natural product of nitrogen and protein metabolism and predominantly found in urine and animal waste. In the aquatic environment, biodegradation of urea is common, releasing carbon dioxide and ammonia. Increasing temperatures, alkalinity, and the presence of the biological enzyme urease can catalyze chemical hydrolysis of urea [2]. Urea has the highest nitrogen content of all solid nitrogenous fertilizers in common use; therefore, more than 90% of the world’s industrial production of urea is used as nitrogen-release fertilizer, and which is expected to increase by 50% towards 2050 to feed a growing global population [3,4,5]. Currently, urea has a large potential market of 180 Mt per year [6].

The above data suggest that dissolved urea is found in groundwater, especially in agricultural areas. It can therefore create favorable conditions for the corrosion of concrete in buildings, but research on the influence of urea on hardened concrete is not very extensive. The latest experiments focus on fresh mixtures with urea and their properties.

For example, the dependence of urea added into a concrete mixture on flowability, heat of hydration, setting time, strength, shrinkage strain, and carbonation resistance was discussed in [7] and [8]. In [9], it is proven that urea has a significant inhibitory effect on early hydration of cement and has almost no effect on the compressive strength of concrete at 28 days of age and lowers water-to-binder ratio, but can also reduce the shrinkage of concrete. An addition of 6% urea acts as a superplasticizer and increases the workability of the mixture, but also increases compressive strength at deep-freeze curing temperatures [10,11].

In [12], it is said that urea itself has no effect on concrete. However, a note referring to deicers says that several deicers, including urea compounds, cause scaling of non-air-entrained concrete, but air-entrained concrete is not attacked. Moreover, due to the possibility of biological decomposition of urea into calcium carbonate, extensive research has been conducted on self-healing concrete in terms of sealing microcracks [13,14,15,16,17]. As is known, urea (CO(NH_2_)_2_), a chemical compound providing reagents for calcium carbonate formation, is among the most commonly used precursors being added to biological culture medium [18,19,20,21,22].

Abdulrasoul [23] examined urea as a corrosion inhibitor for reinforced concrete steel immersed in simulated alkaline pore solution containing 3% NaCl using open-circuit voltage measurements and potentiodynamic polarization technique. The experimental results of this paper showed that urea fertilizer used in 0.2%, 0.5%, and 1% concentrations is an effective inhibitor and gives good corrosion inhibition for concrete-reinforced steel immersed in simulated concrete pore solutions.

Research from the 1960s was generally carried out on steel in contact with solutions of urea and not with concrete. Furthermore, the range of solution conditions over which urea may be regarded as corrosive to steel has not been established [24].

It was also noted that the damaging effects of concrete structures in urea production plants, where hot and moist conditions are common, have been attributed mainly to physical phenomena involving dissolution, penetration, and crystallization of urea within the pores of the concrete [24].

On this basis, it is believed that soil saturated with urea may adversely affect the quality of a building. It can cause efflorescence on the wall or corrosion of concrete in the building’s foundations.

Urea in contact with concrete hydrolyzes with solutions of strong bases, giving ammonia and carbonic acid salt, according to this equation:

NH_2_CONH_2_ + NaOH → NH_3_ + Na_2_CO_3_, (1)

acidifying the concrete environment. A salt with strong base and weak acid is formed, which is hydrolyzed to form light carbonic acid:

Na₂CO₃ + 2H₂O → H₂CO₃ + 2Na⁺ + 2OH⁻.(2)

Sadegzadeh et al. [24] exposed concrete cubes cast from 10 concrete mixes to weekly cycles of drying and submersion in a variety of urea or NaCl solutions for 30 months. The research, which used ultrasonic pulse velocity, compressive strength, mercury intrusion porosimetry, freeze–thaw resistance of concrete, rest potentials, and corrosion rates of embedded steel, indicated no destruction in the urea solutions. None of the specimens showed evidence of significant bulk mechanical deterioration; only surface damage was noted after freezing and thawing cycles. Additionally, no significant corrosion of steel was detected on the specimens exposed to urea solutions. Therefore, it was decided to test the effect of urea using current test methods, like SEM and XRD.

The aim of the work is to understand the effects of urea interaction with a cement binder proposed for the construction of facilities exposed to chemical attack.

## 2. Materials and Methods

### 2.1. Sample Preparation and Aggressive Immersion

The total duration of the experiment was 4 years. Treatment of the sample during the test can be considered simulation of the use of prefabricated elements in agricultural land previously contaminated with urea, and it consisted of aging, immersion in urea solution simulating agricultural groundwater, and natural drying.

A sample of cement paste was made with a water-to-cement ratio equal to 0.4. The chemical composition and other cement data are given in Table 1.

A sample with dimensions of 25 × 25 × 6 cm was cured in water for 6 months. Then the sample was stored under room conditions for 3 years. Therefore, the cement paste was mature. For the next 11 months, the sample was immersed in urea solution under room conditions (T = 20 ± 2 °C). The solution was made of 1 kg of urea (CH_4_N_2_O; solid, pure for analysis, molecular weight of 60.06) and 4 L of deionized water. Thus a 20% solution with a concentration of 4.2 mol_urea_/l_water_ and a pH value of ~9 was prepared. From this, it follows that in the solution acting on the sample with an outer surface of 1850 cm^2^, there were about 16.7 moles of urea. The pH of the solution changed to 9.86 after 7 days, 10.16 after 4 weeks, and 10.26 after 8 weeks. The container was tightly covered to eliminate the perceptible smell of ammonia. Thus, the reaction of ammonium carbonate (urea after hydrolysis according to (3)) with atmospheric carbon dioxide to slightly aggressive ammonium bicarbonate (according to (4)) [24],

CO(NH_2_)_2_ + 2H_2_O → (NH_4_)2CO_3_, (3)

(NH_4_)_2_CO_3_ + CO_2_ + H_2_O → 2NH_4_HCO_3_, (4)

was limited.

According to the hydrolysis of urea,

NH_2_CONH_2_ + 2H_2_O → 2NH_4_+ + CO_3_^2−^ → 2NH_3_ + H_2_CO_3_, (5)

the pH of the environment should slightly decrease because of H_2_CO_3_ production. However, these reactions take place in a highly alkaline environment, so a huge amount of ammonium ions is converted into ammonia, which evaporates. However, buffer reactions cannot be excluded at the site of the reaction as well.

After natural drying for 3 months under ambient conditions, urea crystals destroying the surface of a cement paste sample due to crystallization were not observed, as in [24]. The color and turbidity of the immersion solution did not change either.

The powdered hydrated cement paste was tested. Going deeper into the sample after immersion, powdered slices with a thickness of 0.5, 2, or 4 mm were collected with a diamond head (thickness and depth of the powdered slices and division into subareas shown in Table 2). The grain size of the powder was not greater than 2 micrometers. The ammonia leaked out during abrasion of deeper parts. Each powder layer was stored in a sealed jar. To characterize the samples, the following methods were used. Sub-areas in Table 2 were entered for analysis. The sample tested was named M-1.

### 2.2. pH (Reaction)

The test was conducted on powdered samples. An amount of 100 mL of deionized water with pH 7.0 was added to 10 g of each powdered sample (suspensions of 1:10 mass ratio of powdered paste and deionized water were prepared) and subjected to washing for approximately 5 min up to stabilization of the pH value using a portable pH meter for polluted water.

### 2.3. Conductivity

The test was conducted on powdered samples. Suspensions of 1:10 mass ratio of powdered paste and deionized water (3.28 μS/cm) were prepared. Conductivity was tested after mixing in supernatant water after 1 h, after 1 day, and after 3 days. The conductivity tests were carried out using a CC-505 type conductometer and a CD-3 type conductivity cell probe. The sensor electrodes were plated with platinum covered with platinum black to reduce the polarization phenomena occurring in highly conductive samples. The measuring range of the sensor was from 0.1 µS/cm to 10 mS/cm. For automatic temperature compensation, the set also included a CT2B-121 temperature sensor.

### 2.4. Calcium Content

The test was conducted on powdered samples. Suspensions of 1:100 mass ratio of powdered paste and deionized water (3.28 μS/cm) were prepared and stored at 22 °C. After 24 h, the suspensions were filtered through a filter paper. The calcium content was determined according to the standard PN-EN ISO 11885:2009 for water quality determination of selected elements by inductively coupled plasma optical emission spectrometry (ICP-OES) (ISO 11885:2007).

### 2.5. XRD

For phase composition analysis, an X’Pert Pro MPD X-ray diffractometer, produced by PANalytical (Westborough, MA, USA), was used. The measurements were conducted at room temperature using monochromatic Cu Kα radiation. Qualitative analysis with the support of the ICDD PDF4+ database was performed employing HighScore v. 4.8 software. The test was conducted on powdered samples.

### 2.6. SEM

The morphology of the tested sample was determined using a TESCAN Mira 3 LMU scanning electron microscope equipped with an EDS from Oxford Instruments (Abingdon, UK), supported by Aztec software. The section of sample M-1 was chrome-coated using a Quorum Q150T ES sputter (Quorum Technologies Ltd., Guelph, ON, Canada). The tests were conducted on embedded-in-resin polished material using SEM–BSE operating mode.

## 3. Results and Discussion

### 3.1. pH (Reaction)

**Figure 1:**
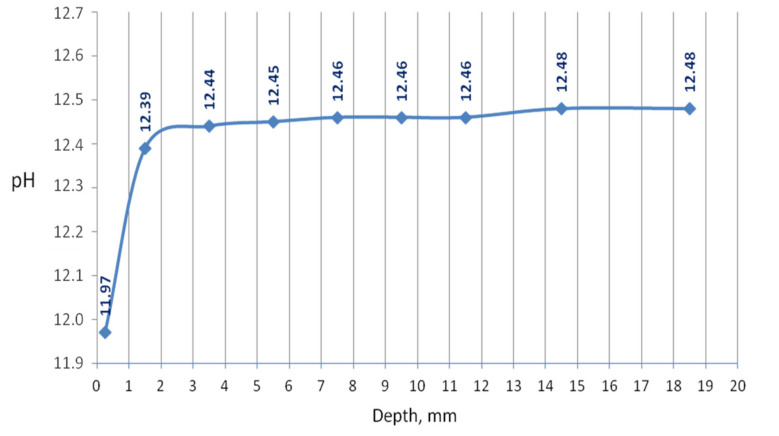
The dependence of pH of hardened cement paste sample after immersion in urea solution on depth.

The penetration of the urea solution caused pH changes in the hydrated cement paste, basically noticeably at a shallow depth at the edge of the sample. Substantially, it can be said that the effect of the solution reaches a depth of 12.5 mm since changes in the pH value have been recorded in the layer up to this depth from the outer surface. However, the 3-month period of natural drying could intensify the homogenization of the chemical conditions in the sample. The pH of the external slice with a thickness of 0.5 mm was 11.97; a pH of a slice located in depth from 0.5 to 2.5 mm was 12.39; pH was equal to 12.44 at a depth of 2.5–4.5 mm; and the deeper, the greater pH until it reached a value of 12.48 at a depth of 12.5 mm. The pH changed drastically in the first slice, which can be seen in the graph in Figure 1.

### 3.2. Conductivity

**Figure 2:**
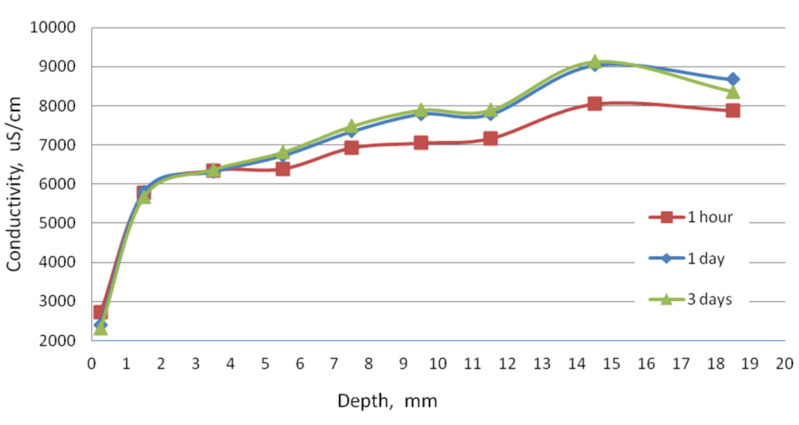
The dependence of conductivity of suspensions of hardened cement paste sample after immersion in urea solution on depth.

The conductivity test was performed to check the condition of the sample in terms of the content of soluble phases. As can be seen in Figure 2, at a depth of 3.5 mm, after 1 day of washing, the phases did not dissolve as much as in the deeper slices of the sample. This demonstrates the slow dissolution of more complex cement hydration compounds and release from the amorphous phase. In the first slice located at a depth to 0.5 mm, the conductivity even decreased, which may be the result of the precipitation reactions. The process of decalcification of the sample took place to a lesser extent, but the resulting changes are evidence of corrosion indeed.

### 3.3. Calcium Leaching

**Figure 3:**
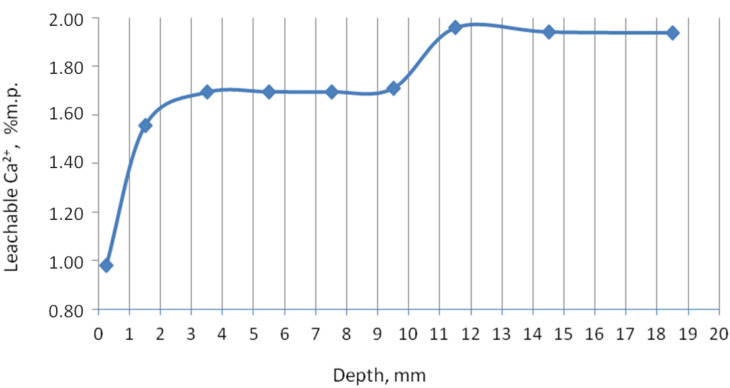
Content of leachable calcium in percentage by mass of hardened cement paste on the depth.

In order to check the content of the calcium phases, which are easily dissolved in water, and which is by default calcium hydroxide, a test was carried out, the results of which are shown in Figure 3. In the edge zone, the content of easily soluble calcium compounds is lowest due to the calcium carbonate formation and lower pH. From about 3.5 to 9.5 mm, the free calcium ion content increased and remained constant, probably released from calcium aluminate silicates and, to a lesser extent, from Ca(OH)_2_. A further increase in calcium ion content and keeping it constant was noted at a depth of approximately 11.5 mm and emerged in addition to other phases, such as portlandite and wollastonite. This test also confirmed the calcium leaching process (i.e., the decalcification of the cement matrix by the action of urea). A similar course for the content of leachable calcium in decalcified samples was presented in [25,26]. Works on the decalcification of the cement paste confirmed the material weakening in general. Although the graph shows a stabilized calcium content deeper than 12 mm, it does not mean that it is healthy material in this zone. Moreover, up to a depth of about 20 mm, the sample was breakable by the force of the hands.

### 3.4. XRD Analysis

**Figure 4:**
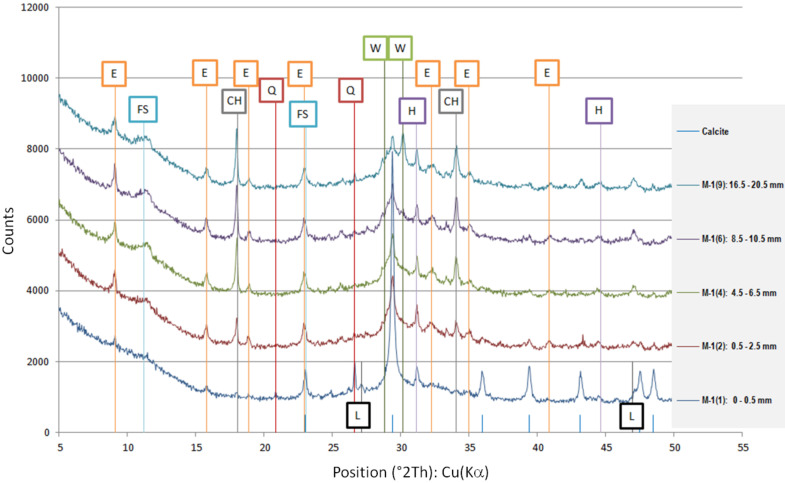
Diffractograms of selected powdered slices of sample M-1 (phases symbols, as in Table 3).

The results of the phase composition tests at different sample depths are presented in Table 3 and Figure 4.

Due to the presence of slag in the cement from which the sample was made, most of the examined powdered slices contained the amorphous phase, which is visible as a diffraction pattern hill around 30 degrees 2 Theta. However, typical slag phases, such as gehlenite or akermanite, were not identified. Among the secondary components, microclines, quartz, and calcium carbonate in the form of calcite were found in trace amounts. Quartz gives the strongest reflection in the outermost powdered slice because it is one of the few crystalline phases here.

The addition of a binding regulator caused the formation of ettringite. Ettringite was the smallest amount in the outermost layer because it probably had decalcified. The same situation occurred with Friedel’s salt, another aluminate, which decomposes during pH drop. In the inner layers, there was a noticeable amount of Friedel’s salt in solid solutions, because the mass of the cement contained about 0.4% chlorine ions (according to an analysis by electrochemical method). Halite was also detected in trace amounts in the inner layers. In outer zone of the sample, NaCl was in greater amount. Probably halite formation benefited from the decomposition of Friedel’s salt.

The sample was carbonated. This could be easily traced by looking at the 100% calcite reflex at the 2 Theta angle around 29.4, which decreased with increasing depth. This means that free calcium was available (i.e., the phases of the hardened paste were gradually decalcified).

A large amount of wollastonite, identified in highest amount in powdered slice 9 (named M-1(9)) at a depth of 16.5–20.5 mm, decreased towards the edge of the sample, but its reflex was still noticeable at a depth of 10.5 mm (100% intensity at 30.150 °2Th). Wollastonite CaSiO_3_ is a ribbon silicate consisting of [SiO_3_]^2−^ groups and Ca^2+^ cations. The task of Ca^2+^ cations is to bond the silica ribbons into a spatial lattice of their crystals [27]. Therefore, since there was less of this crystal in the diffractogram of powdered slice 6 (named M-1(6)), it can be concluded that we have evidence of decalcification at a depth of about 10 mm. It was surprising that Lepidocrocite FeO(OH) was also detected in the boundary layer. This phase is present in the natural environment, but can also come from metallurgical processes after oxidation, which is more possible in the case of specimen M-1.

### 3.5. SEM Analysis

The sample was characterized by low strength up to a depth of 17 mm, which could indicate a certain front of material weakening. The observed microstructure of the polished section of sample M-1 was relatively compact, with low internal porosity. The locally visible pores were spherical in shape, with diameters up to approximately 100–500 μm. The microstructure of the material consisted of irregular grains of unreacted slag “embedded” in a relatively uniform, locally porous matrix of hydrated cement paste. The size of the observed grains ranged widely from 100 µm to several micrometers.

**Figure 5:**
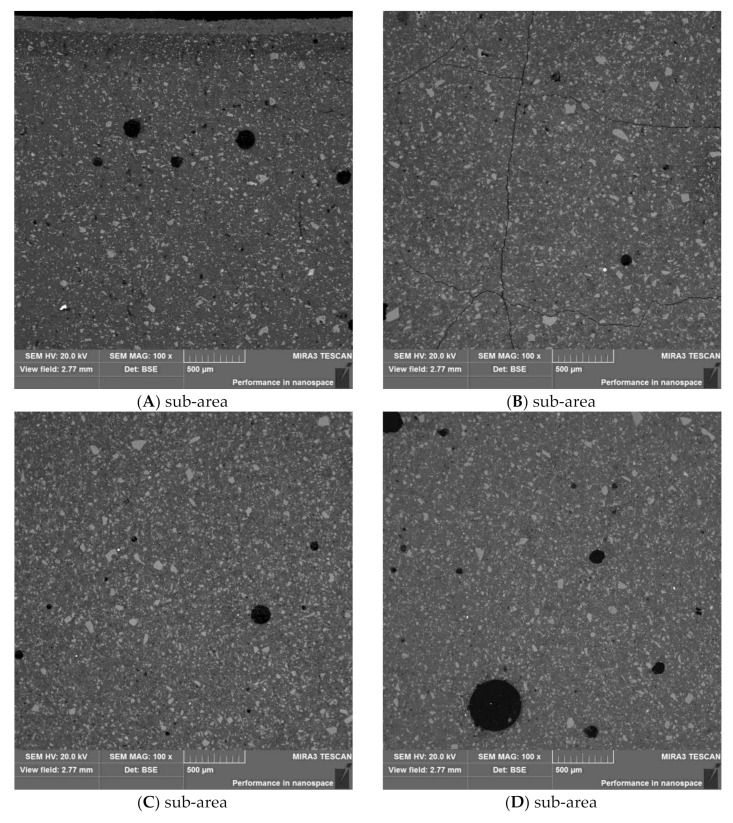
Comparison of the microstructures of (**A**), (**B**), (**C**), and (**D**) sub-areas.

Initial observation of the specimen microstructure imposed a division into four sub-areas, marked A, B, C, and D, as described in Table 2 and shown in Figure 5. From the images, it can be concluded that the smallest number of grains was in sub-area A. Sub-area B, on the other hand, showed the highest amount of microcracks.

**Figure 6:**
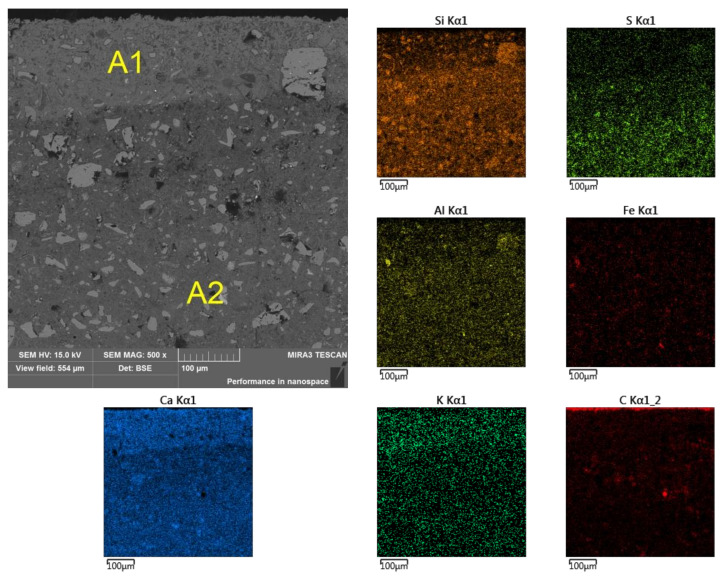
Division of sub-area A into zones A1 and A2 and elemental surface analysis (selected elements).

**Figure 7:**
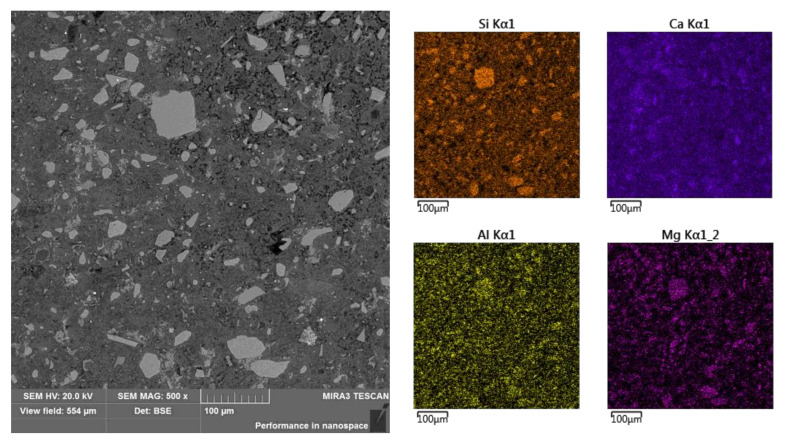
Sub-area B and elemental surface analysis (selected elements).

**Figure 8:**
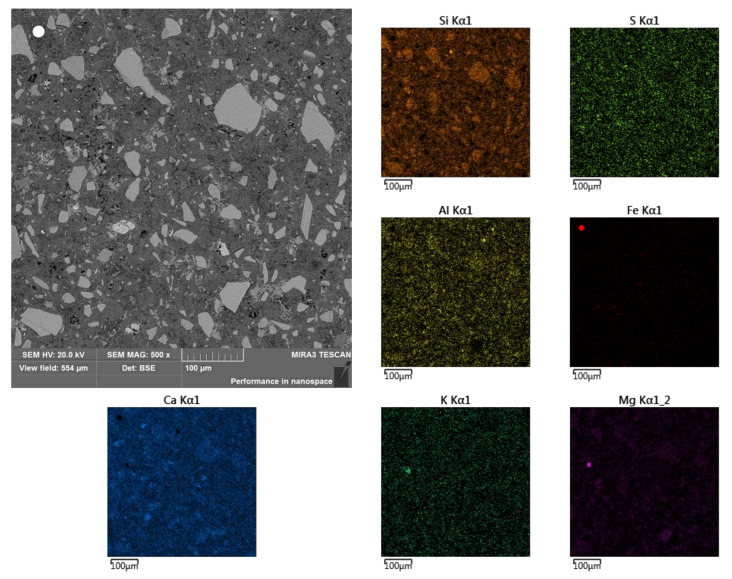
Sub-area C and elemental surface analysis (selected elements).

**Figure 9:**
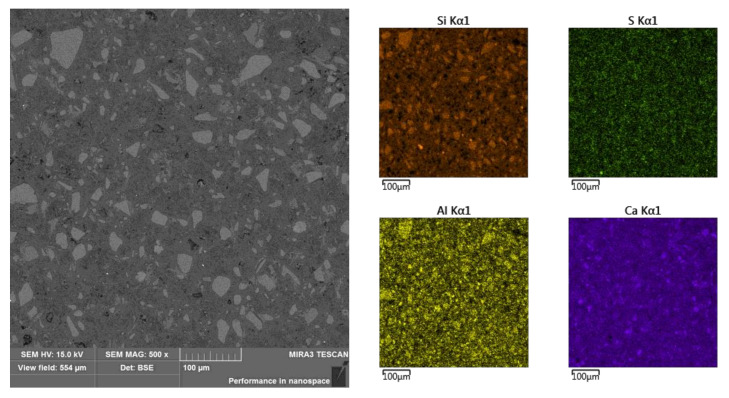
Sub-area D and elemental surface analysis (selected elements).

Observation of the sample’s cross section revealed the occurrence of layered material, especially in micro-area A, for which, from the side of the outer edge, a layer of approximately 140 μm was marked as A1 with a lighter shade (Figure 6). Mapping the chemical composition of the A1 area showed a reduced content of aluminum, silicon, and sulfur with a simultaneous increased content of potassium and calcium, compared with the other analyzed micro-areas (i.e. A2, B, C, and D (Figure 7, Figure 8 and Figure 9 and Table 4)).

#### 3.5.1. Sub-Area A Analysis

**Figure 10:**
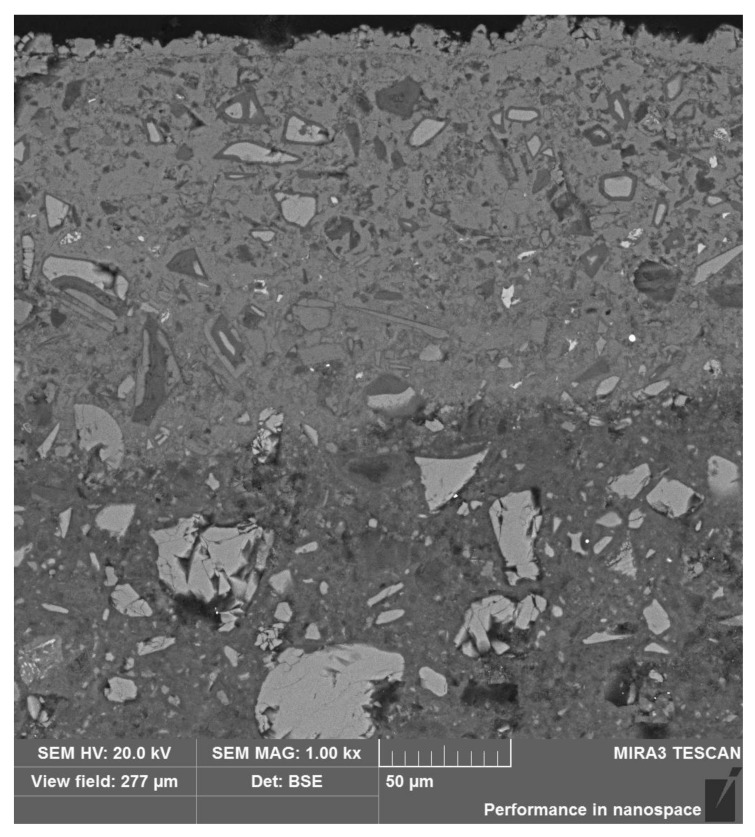
The visible border of the front at a depth 140 μm between zones A1 and A2.

In the lighter shade of the outermost zone A1, the slag grains had darker rims, indicating their pozzolanic reactions and probably reactions with urea (Figure 10). Moreover, in the mass of the A1 sub-area, amorphous grains of calcium carbonate were present, and the remaining grains were calcium and magnesium silicoaluminates, calcium silicates in solutions constituting melilities. They were accompanied by traces of phosphorus, chlorine, titanium, manganese, sodium, and sulfur. Occasionally, potassium and chromium could be seen in higher amounts. On the outermost part of the A1 sub-area, a separate layer was formed: loosely bound, called “skin” (Figure 10). “Skin,” frayed and with a medium thickness of 10 μm, consisted of calcium carbonate.

The grain was also examined and located in zone A1, which was divided into three shades (left part of the image in Figure 10 and Table 5). In direction from the center to the surface of the grain, the contents of aluminum, silicon, and potassium decreased, and the content of calcium increased. Titanium, manganese, magnesium, iron, and sulfur were found in greater amounts in the middle and outer parts of the grain. The inner zone was characterized by the highest contents of silicon, aluminum, magnesium, and potassium. The composition of the intermediate layer indicates the occurrence of pozzolanic reactions (increased Ca/Si ratio), and the outermost layer indicates the influence of urea. This is explained by the fact that the amount of carbon in relation to silicon increased towards the grain envelope (Table 5).

In Table 6, where the image of the micro-area is shown, it can be seen that in zone A1 can be found grains with lighter or darker shells than those in the interior of the grain. The analyses in points S1–S4 confirm the reactivity of the grains with the components of the binder matrix. There is less calcium in the shell of the grains—it was most likely washed away and reacted with carbon dioxide. However, in the analysis of the binder matrix in the A1 and A2 zones, points S5 and S6 confirm the presence of a large amount of calcium (in the form of carbonate) and the typical composition of the slag binder, respectively.

**Figure 11:**
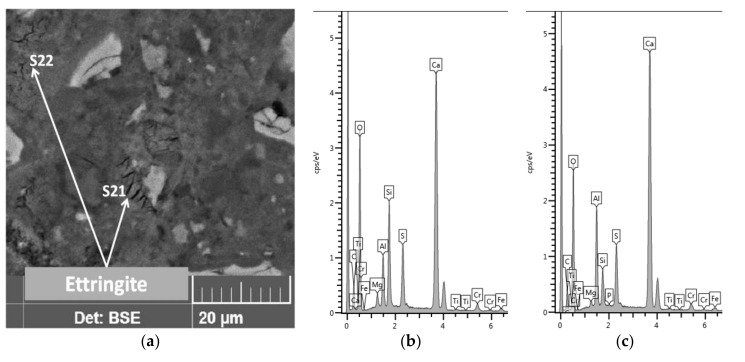
Ettringite (**a**) and its spectra: (**b**) S21 and (**c**) S22, area A2.

Another light grain seen in zone A1, which was not shelled but had very high aluminum (Al/Si = 3.8) and iron (Fe/Si = 4.9) contents with low masses of oxygen (O/Ca = 1.5) and silicon (Si/Ca = 0.08). A grain with an equally light shade, due to the iron content, was also present in zone A2 (Table 7, analysis point S15). Thus, the variation among binder grains was quite large. Ettringite, on the other hand, created cracked, shrunken, stratified, dehydrated grains (Figure 11).

#### 3.5.2. Sub-Area B Analysis

**Figure 12:**
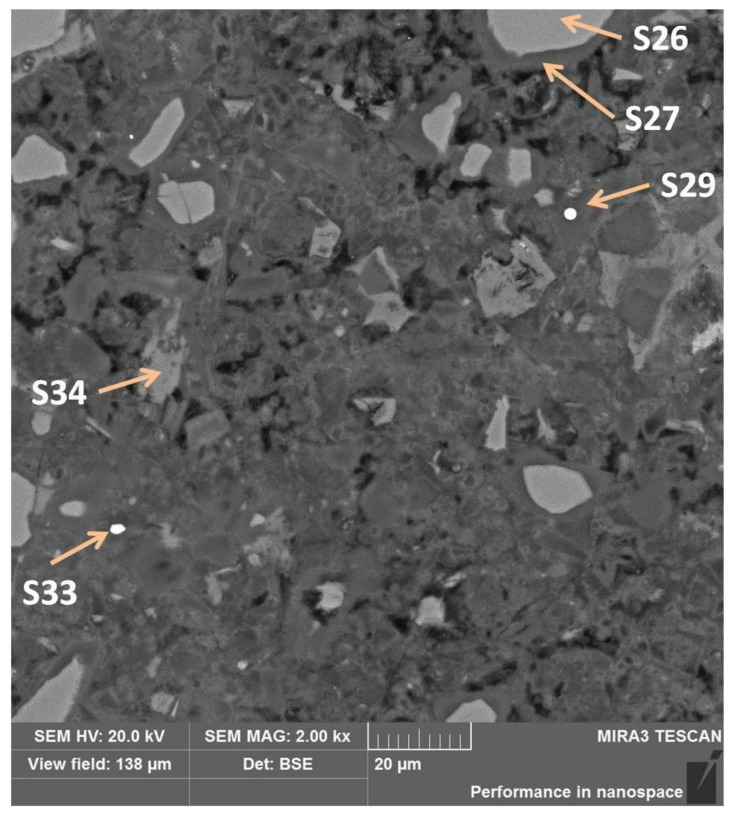
Sub-area B with visible high-porosity zones and selected EDS test points.

In sub-area B, places of high porosity are visible (Figure 12), which may indicate that the penetrating urea solution had the ability to dissolve the binder. Moreover, in contrast to the A sub-area, the pores in the B sub-area had not yet managed to “overgrow” with a sufficient amount of corrosion products, mainly secondary calcite. A very similar way of passing the degradation fronts was observed in the corroded hardened cement paste after immersion in ammonium chloride solution, as a result of which ammonia was also released [28].

By analyzing the composition of an exemplary grain with a lighter shade than its shell (measurement points S26 and S27 (Figure 12 and Table 8)), it can be concluded that the contents of calcium and magnesium increased and the content of aluminum decreased in relation to silicon, moving from the center to the grain shell. Therefore, Mg might have a higher affinity for the exit from the slag grain matrix, assuming that the silicon–oxygen bond was least resistant to disintegration. The amount of oxygen in the shell also increased from 34% to 47% in relation to the total weight of the sample.

In measurement point S34, a phase with a light irregular shape without a shell, filling the free space of the pores, it is calcium carbonate formed in the reaction of free calcium (from phase decalcification) with an CO_3_^2−^ ion (from urea hydrolysis). This conclusion results from the calculations presented in Table 8.

The phases with the S29 and S33 measurement points were characterized by a lighter shade of gray. In Table 9, their spectra are shown. Both have a large amount of iron, but due to the fact that chromium was not taken into account in the analysis, because the sample was sputtered with this element, the iron content is overestimated. In S33, it is 49.2%, while in S29, iron is about 64.6%. In S33, there is also a metallic phase, but with a very high chromium content. The metallic phases come from the alloys contained in the slag.

#### 3.5.3. Sub-Area C Analysis

**Figure 13:**
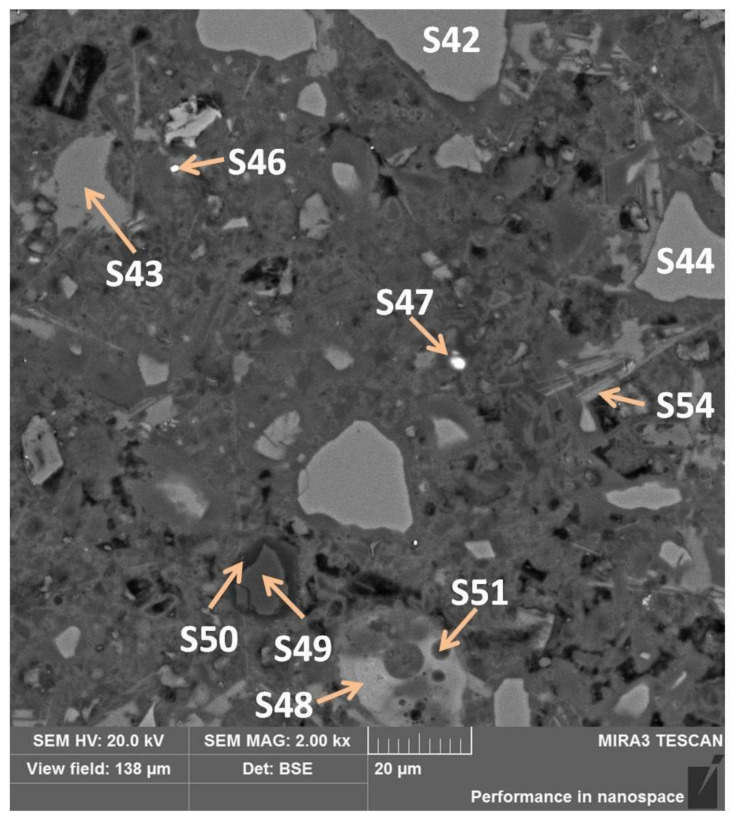
Sub-area C with selected EDS test points.

Sub-area C is also microstructurally rich in various phases. There are light grains with darker shells, lamellar or stick phases, pores, and slag sinters (Figure 13).

In Figure 13, slag grains were marked S42 and S44 and slag sinter, which was examined in points S48 and S51. The difference between them is in the microstructure: in S42 and S44, no Fe was identified, but there were much more Mg, more Si, less K, and less Ca (Table 10). Looking at the sinter, in the lighter zone (S48), there were more oxygen, calcium, and iron and less carbon and phosphorus appeared; and in the dark (S51), zinc. Another grain, tested in points S49 and S50 located in its shell, contained mainly magnesium in oxidized form or its alloy (Table 10). Glowing points S46 and S47 were iron alloys (15% and 47%). The phase marked with point S43 contained the highest amount of calcium, which most probably is amorphous calcium hydroxide. Another phase with a large amount of calcium was examined in S54, which, in turn, may be crystalline calcium hydroxide in the form of a package of parallel-arranged “cards” [28], and the remaining elements (Table 10) are its surrounding.

#### 3.5.4. Sub-Area D Analysis

**Figure 14:**
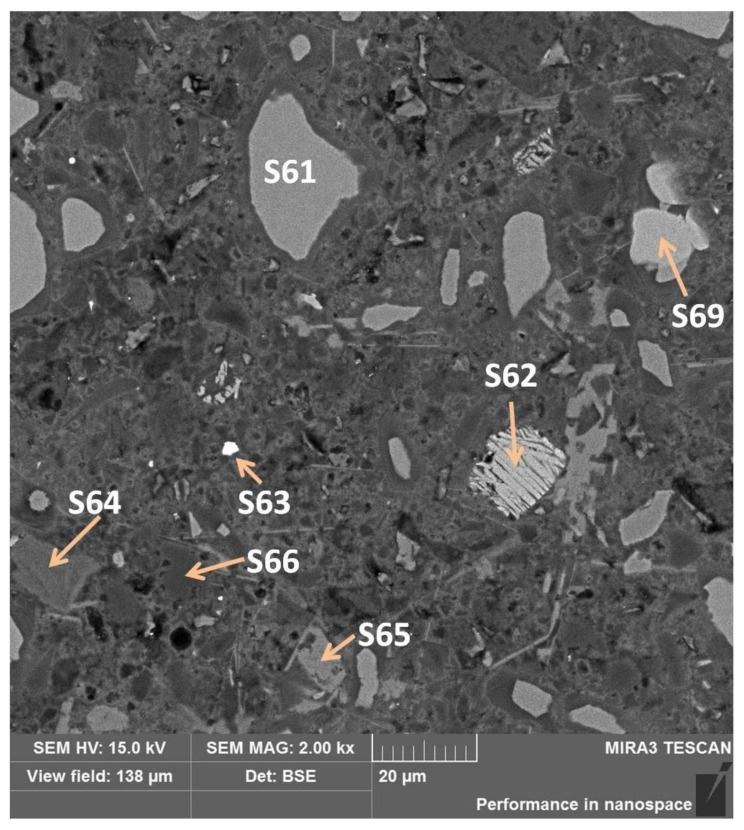
Sub-area D with selected EDS test points.

Sub-area D with high phase richness was chemically examined at the points shown in Figure 14. As before, amorphous calcium hydroxide took the form of a phase filling the free space, point S65 (Table 11). The inclusions of metallic phases also occurred in this subarea, as shown in S63 (Table 11) as a metallic iron phase. The darkest shade of gray among the grains was found in the S66 test point, which was SiO_2_, silica (Table 11). The previously unseen grain with a cracked layer morphology (S62) consisted mainly of oxygen, calcium, and silicon (Table 12), where Ca/Si = 2.7. Therefore, it could be C_3_S_2_ or C_2_S found in metallurgical slags [29]. However, if we assume that silicon is slightly below the test crystal, we probably see portlandite here. Comparing the results of microanalytical tests in point S69 with points S61 and S64 (Table 12) of the grains, the brightest phase determined by the analysis of S69 indicates akermanite (C_2_MS_2_), merwinite (C_3_MS_2_), or monticellite (CMS). A typical grain described in S61 is calcium silicate enriched with aluminum and magnesium and other trace elements (Table 12) forming solid solutions in the smelting process. On the other hand, the darkest S64, compared with S61, contained more oxygen, traces of aluminum, and no magnesium (Table 12).

**Figure 15:**
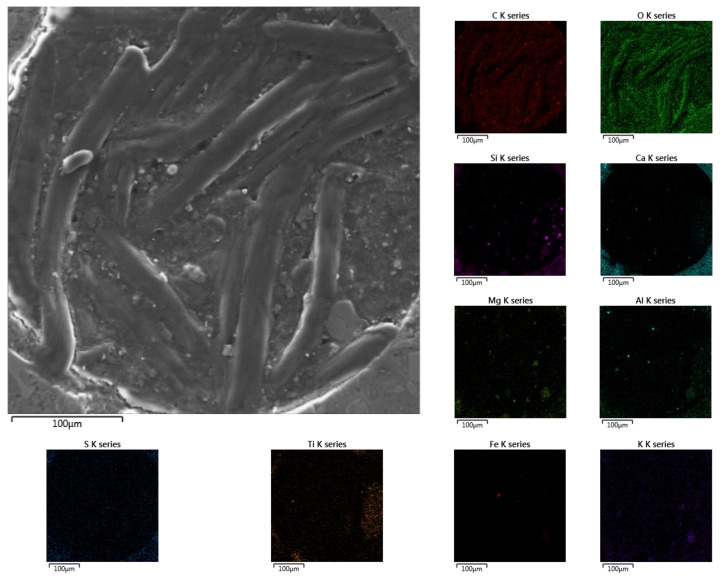
The interior of the pore filled with wormlike structures in zone D with element distribution maps.

Very interesting, however, seems to be an analysis of what was found in the air pore in the D sub-area (Figure 15). The twisted wormlike stamens were predominantly composed of carbon and oxygen, suggesting the presence of organic compounds in the pore. Perhaps even life developed in it, under the influence of the life-giving urea.

Taking into account the obtained test results, then it is necessary to look into the crystalline phases formed at the fracture. Future research directions may also focus on using other types of cement. It also seems necessary to extend the experiments to the diagnosis of decalcified concrete in the environment of buffers giving erroneous interpretations of the degree of concrete corrosion. The research should also be extended to check microhardness depending on the depth in the sample.

## 4. Conclusions

This article returns to the issues of concrete corrosion caused by urea, because in recent years this problem has been to some extent forgotten.

The research methods used in this article showed that after almost a year of immersion in 20% urea solution of hardened cement paste made of CEM III/A 42,5 N-LH/HSR/NA,

- the pH of the outer layer with a thickness of approximately 1 mm decreased by about 0.5 units
- the outer 20 mm layer was apparently changed chemically, in phase and consequently mechanically
- a 10 μm skin of calcium carbonate was formed on the surface of the sample
- in the outermost layers (up to 3.5 mm), there were not too many cement hydration complex phases soluble in water
- in the boundary zone up to 1.5 mm were not present the water-soluble calcium phases
- the presence of gaseous ammonia was noticeable in the entire sample, which indicates a reaction of urea with the hardened cement paste phases
- the slag grains had a shells with a different chemical composition and the change in the composition was not gradual
- the dominant crystalline phase was secondary calcite in the edge zone of the sample
- the largest amounts of wollastonite were located 16.5 mm deeper, and up to this depth, decalcification surely took place.

Moreover, while urea diffuses through concrete, there is a possibility of forming buffer solutions in micro-areas. For these studies, it could be an ammonium or/and bicarbonate buffer. Therefore, in this situation, pH cannot serve as a concrete corrosion indicator.

## Figures and Tables

**Table 1:** Characteristics of CEM III/A 42,5 N-LH/HSR/NA.

Index	Unit	Value	Description
Rs2	MPa	14.9	Cement CEM III/A 42,5 N-LH/HSR/NA, is a slag cement that has a strength class of 42.5 MPa, has normal early strength (N) with low heat of hydration (LH), is resistant to sulfates (HSR), is low alkaline (NA), and has composition in accordance with the requirements of the standards PN-EN 197-1 and PN-B-19707. The use of this cement, among others, minimizes the risk of thermal stresses in massive structures and increases the corrosion resistance of mortars and concretes. Cement Monolit 42.5 with dosing in accordance with the European standard PN-EN 206 and Polish PN-B-06265 can be used in all exposure classes. It consists of a slag with 50–65%; OPC: 35–50%; 5% is a secondary additive and setting time regulator in the form of calcium sulfate. Slag cements are commonly used in Poland due to the steel industry (slowly disappearing), and the use of slag may become unprofitable. The slag gives cement many favorable properties. In the exemplary year of 2017, more than 2 million tonnes of slag was used for the production of cement.
Rs28	MPa	57.6	
H_2_O	%	31.6	
Sti	min	238	
Stf	min	293	
SSB	cm^2^/g	4760	
SiO_2_	%	29.08	
Al_2_O_3_	%	6.30	
Fe_2_O_3_	%	1.37	
CaO	%	48.82	
MgO	%	4.36	
SO_3_	%	2.74	
Na_2_O	%	0.34	
K_2_O	%	0.73	
eqNa_2_O	%	0.82	

**Table 2:** Powdered slices and sub-areas.

Name	M-1(1)	M-1(2)	M-1(3)	M-1(4)	M-1(5)	M-1(6)	M-1(7)	M-1(8)	M-1(9)
**Depth, mm**	0–0.5	0.5–2.5	2.5–4.5	4.5–6.5	6.5–8.5	8.5–10.5	10.5–12.5	12.5–16.5	16.5–20.5
**Thickness, mm**	0.5	2	2	2	2	2	2	4	4
**Sub-areas**	A	B							C

Sub-area D deeper than 25 mm.

**Table 3:** The depths of the occurrence of phases.

Layer: Depth, mm	Amorphous Phase	CH—Portlandite	C—Calcite	E—Ettringite	Q—Quartz	FS—Hydrocalumite	H—Halite	L—Lepidocrocite	W—Wollastonite
M-1(1): 0–0.5	main		✓	✓	✓	✓	✓	traces	
M-1(2): 0.5–2.5	main	✓	✓	✓	✓	✓	✓		✓
M-1(4): 4.5–6.5	main	✓	✓	✓	✓	✓	✓		✓
M-1(6): 8.5–10.5	main	✓	✓	✓	✓	✓	✓		✓
M-1(9): 16.5–20.5	main	✓	✓	✓	✓	✓	✓		✓

**Table 4:** Chemical composition of the micro regions of the M-1 sample based on the EDS mapping of the chemical composition.

Area	Ingredient Content, wt.%											
	C *	O	Na	Mg	Al	Si	S	K	Ca	Ti	Mn	Fe
A	15.6	47.4	0.2	1.9	2.2	8.8	0.8	0.2	22.1	0.1	0.1	0.7
B	8.8	51.0	0.3	1.8	2.3	9.3	1.1	0.5	24.0	0.1	0.1	0.7
C	5.5	51.0	0.3	2.1	2.4	10.3	1.1	0.5	25.6	0.2	0.1	0.8
D	4.9	51.9	0.3	2.0	2.4	10.4	1.2	0.6	25.5	0.1	0.1	0.6

* The carbon content is affected by the phenomenon of sample contamination, and in the case of a micro-area, increased C content is also influenced by the presence of resin in the analyzed micro-area.

**Table 5:** Elemental composition of the slag grain with the division into 3 zones in the A1 subzone.

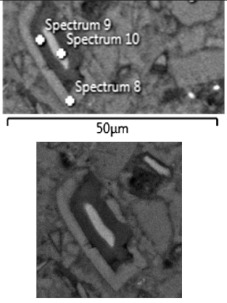	**Element**	**Spectrum 8**	**Spectrum 9**	**Spectrum 10**
	**C**	21.08	24.51	18.59
	**O**	46.79	45.01	47.67
	**Na**	0.20	0.25	0.16
	**Mg**	0.85	0.27	1.01
	**Al**	0.92	2.08	3.73
	**Si**	3.66	6.34	14.27
	**S**	0.03		0.14
	**K**	0.39	1.58	1.72
	**Ca**	25.02	19.44	11.45
	**Ti**	0.47	0.33	0.44
	**Mn**	0.34		0.49
	**Fe**	0.25	0.19	0.33
	**Ca/C**	1.18	0.79	0.62
	**Ca/Si**	6.84	3.07	0.80
	**C/Si**	5.76	3.87	1.30

**Table 6:** Characteristics of zone A, selected elements.

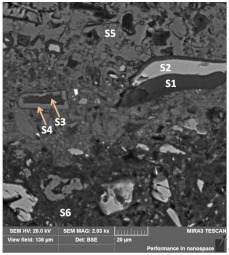	**Point**	**Ca/Si**	**Al/Si**	**Mg/Si**	**O, %**	**Ca, %**
	**S1**	0.54	0.29	0.2	50.8	10.7
	**S2**	1.58	0.2	0.24	46.5	24.8
	**S3**	20.9	0.39	0.02	51.7	21.7
	**S4**	1.62	0.11	0.07	54.5	18.6
	**S5**	80.00	0.17	0.00	50.0	36.1
	**S6**	1.83	0.24	0.27	53.6	18.6

**Table 7:** Chemical analyses of EDS and spectra at points in grains in zone A2.

El.	S15		S24	
**C**	12.54	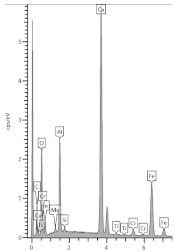	20.71	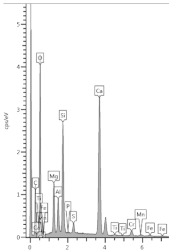
**O**	32.25		49.26	
**Mg**	0.42		3.88	
**Al**	7.69		2.32	
**Si**	0.33		6.85	
**Ca**	27.29		15.54	
**Ti**	0.27		0.17	
**Fe**	19.21		0.3	
			P: 0.06 S: 0.75 Mn: 0.18	

**Table 8:** The spectra and the ratios of the contents of the elements in individual points of the B sub-area in Figure 8.

Point	S26 (Interior Grain)	S27 (Exterior Grain)	S34 (Calcium)
Spectrum	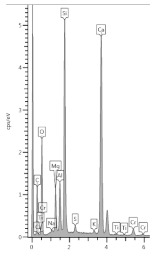	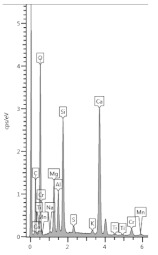	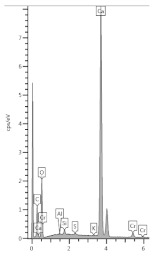
Ca/Si	1.73	2.00	93.80
Al/Si	2.45	0.38	Ca/O = 0.9
Mg/Si	0.22	0.55	Ca/C = 3.18

**Table 9:** The spectra and the ratios of the elements’ contents at individual points in Figure 12.

Point	S29 (Fe)	S33 (Cr)
Spectrum	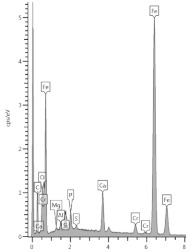	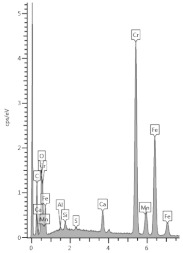

**Table 10:** The mass percentage of elements in individual measuring points shown in Figure 11.

Element	S42	S44	S48	S51	S49	S50	S43	S54
**C**	31.59	30.13	26.55	38.98	26.92	25.88	17.65	24.28
**O**	33.35	32.91	31.96	24.81	36.23	47.12	40.40	40.84
**Na**	0.19	0.21	0.37	0.39		0.17		0.31
**Mg**	3.52	2.67	0.60	0.40	34.76	22.79		0.30
**Al**	2.08	2.21	3.36	2.10		0.21		0.48
**Si**	11.87	12.08	8.95	9.72	0.11	0.93	0.27	2.26
**P**			0.15					
**S**	0.48	0.46	0.77	0.71	0.05	0.09	0.06	0.51
**K**	0.40	0.59	1.58	2.42				0.27
**Ca**	16.02	18.30	21.76	18.52	0.29	1.66	41.63	30.49
**Ti**	0.51	0.24	0.22	0.06				0.07
**Mn**		0.20						
**Fe**			3.73	1.35	0.65	0.39		0.19
**Zn**				0.54	0.99	0.75		

**Table 11:** Spectra and contents of selected elements at measuring points shown in Figure 14.

Point	S65	S63	S66
Spectrum	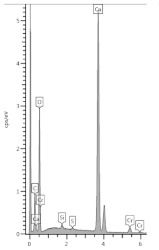	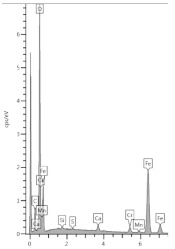	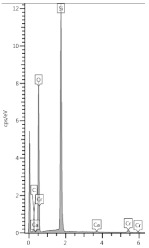
O, %	46.6	31.6	47.3
Ca, %	45.6	1.3	0.3
Si, %	0.4	0.2	32.2
Fe, %	-	56.8	-
C, %	7.3	9.4	20.2

**Table 12:** The percentages of the elements in the individual measuring points shown in Figure 14.

Element	S62	S69	S61	S64
C	11.56	12.88	17.24	15.73
O	38.82	36.34	36.80	48.04
Na			0.24	0.28
Mg	0.10	2.97	3.23	
Al	0.02		3.27	0.87
Si	13.14	13.88	14.85	12.53
S			0.43	0.28
K	0.22		0.30	0.92
Ca	36.13	33.93	23.63	21.36
